# Terroir Influence on Polyphenol Metabolism from Grape Canes: A Spatial Metabolomic Study at Parcel Scale

**DOI:** 10.3390/molecules28114555

**Published:** 2023-06-05

**Authors:** Kévin Billet, Sébastien Salvador-Blanes, Thomas Dugé De Bernonville, Guillaume Delanoue, Florent Hinschberger, Audrey Oudin, Vincent Courdavault, Olivier Pichon, Sébastien Besseau, Samuel Leturcq, Nathalie Giglioli-Guivarc’h, Arnaud Lanoue

**Affiliations:** 1 EA 2106 “Biomolécules et Biotechnologies Végétales”, UFR des Sciences Pharmaceutiques, Université de Tours, 31 av. Monge, F-37200 Tours, France; kevin.billet@inrae.fr (K.B.); thomas.dugedebernonville@limagrain.com (T.D.D.B.); audrey.oudin@univ-tours.fr (A.O.); vincent.courdavault@univ-tours.fr (V.C.); olivier.pichon@univ-tours.fr (O.P.); sebastien.besseau@univ-tours.fr (S.B.); nathalie.guivarch@univ-tours.fr (N.G.-G.); 2 INRAE, UR1268 BIA, Team Polyphenol, Reactivity & Processing (PRP), F-35653 Le Rheu, France; 3 GéoHydrosystèmes Continentaux (GéHCO), EA 6293, Université de Tours, F-37200 Tours, France; salvador@univ-tours.fr (S.S.-B.); florent.hinschberger@univ-tours.fr (F.H.); 4 Limagrain, Centre de Recherche, Route d’Ennezat, F-63720 Chappes, France; 5 Institut Français de la Vigne et du Vin, F-37400 Amboise, France; guillaume.delanoue@vignevin.com; 6 Laboratoire CITERES, Equipe Laboratoire Archéologie et Territoires (LAT), UMR 7324 CNRS, Université de Tours, F-37200 Tours, France; samuel.leturcq@univ-tours.fr

**Keywords:** metabolomics, spatialization, terroir, grape, polyphenols, correlation network, vineyards

## Abstract

The composition of bioactive polyphenols from grape canes, an important viticultural byproduct, was shown to be varietal-dependent; however, the influence of soil-related terroir factors remains unexplored. Using spatial metabolomics and correlation-based networks, we investigated how continuous changes in soil features and topography may impact the polyphenol composition in grape canes. Soil properties, topography, and grape cane extracts were analyzed at georeferenced points over 3 consecutive years, followed by UPLC-DAD-MS-based metabolomic analysis targeting 42 metabolites. Principal component analyses on intra-vintage metabolomic data presented a good reproducibility in relation to geographic coordinates. A correlation-driven approach was used to explore the combined influence of soil and topographic variables on metabolomic responses. As a result, a metabolic cluster including flavonoids was correlated with elevation and curvature. Spatial metabolomics driven by correlation-based networks represents a powerful approach to spatialize field-omics data and may serve as new field-phenotyping tool in precision agriculture.

## 1. Introduction

Grape polyphenols represent a broad range of bioactive specialized metabolites whose variation depends on genotype and environmental conditions. They are highly diverse and distributed as stilbenoids, flavonoids, and phenolic acids. Stilbenoids are important grape phytoalexins that prevent attacks by fungi, bacteria, nematodes, and herbivores [1]. Stilbenoids are largely accumulated in grape canes, an abundant viticultural byproduct [2]. *E*-resveratrol and its oligomeric derivatives are well-studied biomolecules thanks to their health benefits, including the prevention of several diseases of particular concern such as cancers, obesity, atherosclerosis, and Alzheimer’s disease [3]. Grape polyphenols are also involved in cell longevity and health maintenance [4] and act as a cardiovascular protectant in early atherosclerosis [5]. Biocontrol activity of grape cane extracts towards grape downy mildew was also reported [2].

Grape polyphenol metabolism is highly plastic and relies on interactions between genotypic and environmental factors. Polyphenol-based discrimination within genotypes of the same berry color has been reported for berries [6] and wines [7], as well as in grape byproducts [2]. Additionally, environmental conditions may also alter polyphenol composition. Indeed, plant water status and sunlight exposure are important regulators of flavonoids in berries [8], and disease pressure observed during the growing season was shown to impact the polyphenol composition in grape cane harvest during winter [9].

The scientific interpretation of the terroir concept reflects the complex interaction between grapevine, topography (slope, curvature, elevation), climate (temperature, rainfall, and solar radiation), soil properties (pedology, geology, and microbiome), as well as viticultural and wine-making practices [10]. Whereas it was possible to show regional variations in the chemical composition of grape and wine, understanding the determinant factors that drive grape quality remains highly challenging because terroir expression is the result of a multi-parametric system that must be considered at the within-vineyard scale.

Several “omics” approaches were successively deployed to investigate the complexity of terroir signature in distant vineyards [11]. Indeed, metabolomics was used to reveal the signature of terroir and vintage in wine and grape quality [12,13]. In addition, transcriptomics revealed the metabolic plasticity of berries from three macro-areas in the region of Verona in Italy [14]. Finally, metagenomics highlighted the effect of regional microbial diversity on wine quality [15], and variability in epigenetics was also observed in different geographic locations [16]. Despite these recent advances, no “omics” study tackles the terroir influence in continuous space with readily assessable environmental changes. The measurements of georeferenced positions enable spatial monitoring of soil properties and grapevine response [17]; therefore, the spatialization of metabolomics datasets appears to be a meaningful strategy to investigate terroir influence. Additionally, correlation-based networks are useful to explore and interpret complex datasets through the identification of key network nodes [18]. They have been used to reveal grape polyphenols with similar patterns of accumulations under genotypic variations and following pathogen infection [2].

The aim of this study was to assess soil- and topography-based terroir influence in a vineyard parcel covering different soil types with a contrasted topography and planted with a unique clone of Cabernet Franc located in the Loire Valley (France). Soil properties and topography were analyzed at georeferenced points using spatial coverage sampling and grape canes were harvested at the same positions over 3 consecutive years prior to UPLC-DAD-MS-based metabolomics analysis. Principal component analyses revealed vintage and intra-vintage effects. Correlation-based networks were used to assess the global structure of the metabolomic datasets in relation to topography and soil properties over the 3 years. Finally, the conserved metabolomics clusters were mapped and compared to soil property maps.

## 2. Results

### 2.1. Soil Properties and Topography Revealed Contrasted Zones in the Vineyard

**Figure 1:**
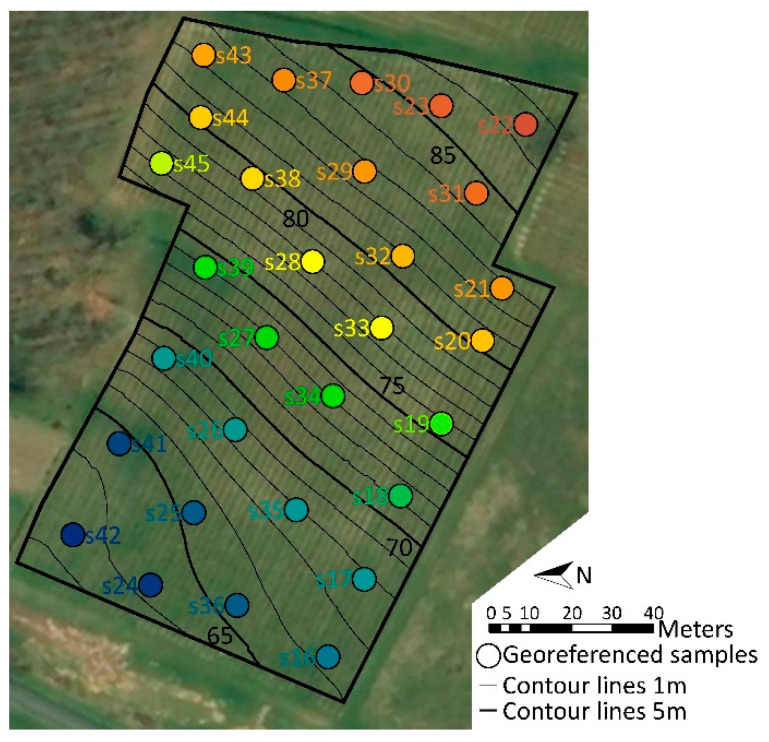
Vineyard located in the commune of Rivière (Loire Valley, France). Grape canes and soil samples were harvest at 30 georeferenced points with a spatial coverage sampling strategy (colored circles). Colors corresponded to elevation.

The texture of the soil (0–30 cm depth) was analyzed at 30 georeferenced points with a spatial coverage sampling strategy (Figure 1) and characterized using the USDA texture triangle (Figure S1). Three positions corresponded to sand (Sa: s22; s30; s31), five to loamy sand (LoSa: s23; s24; s28; s29; s32), a large number of positions to sandy loam (SaLo: s16–s20; s25; s27; s33–s42; s45), and four positions to sandy clay loam (SaCILo: s21; s26; s43; s44). The vineyard (150 m length, 92 m width; 1.35 ha), with an elevation ranging from 62 to 87 m, was orientated west-south-west with an average slope of 14% and a maximal slope of 24% at mid-slope, thus expressing a convex–concave shape (Figure S2).

The subsoil is characterized by upper Cretaceous deposits. The upper convex part of the vineyard mostly consisted of sandy soils developed from non-carbonated, sandy deposits. The middle part with high slope values consisted of carbonated soils developed from Turonian chalks. The lower, concave part of the vineyard consisted of soil developed from colluvial material. The particle–size distribution partly reflected this soil distribution, with more coarse sand in the topsoil horizons located at the top of the vineyard, whereas fine and coarse silts were more abundant in the topsoil horizons at the bottom of the vineyard (Figure S3). Low soil organic matters and nitrogen contents were found upslope on sandy soils, whereas soils rich in organic matter, organic carbon, and nitrogen were found in the slope and downslope, corresponding to soils derived from the chalk and colluvial deposits (Figure S4).

### 2.2. Metabolomics Study of Grape Canes over the Three Vintages

**Figure 2:**
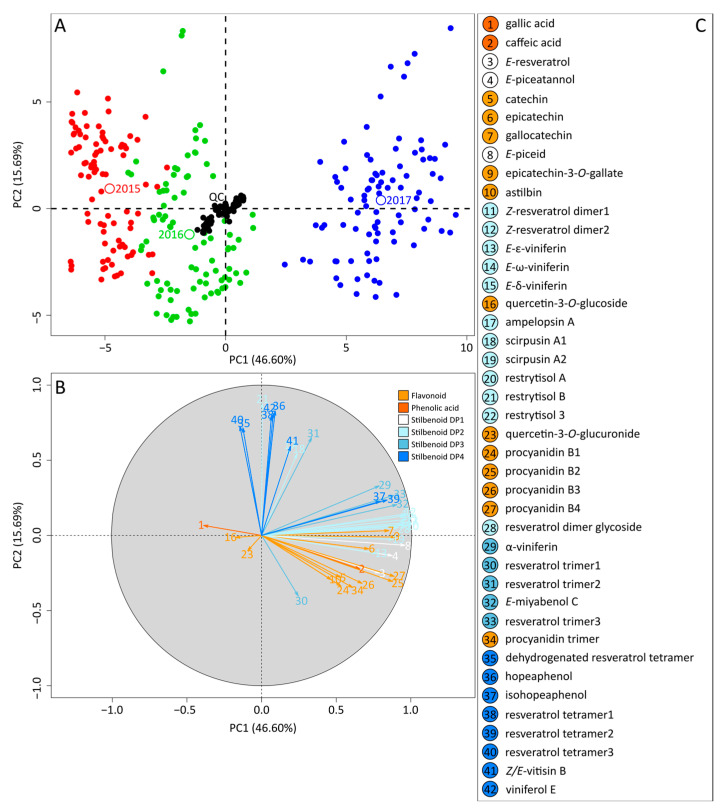
PCA of metabolomics data over 3 years. Variables in score plot (**A**) were colored according to vintages and QC samples. Variables in loading plot (**B**) were colored according to polyphenols’ class and numbered according to compound name (**C**).

A targeted analysis of grape cane metabolome was performed for the 30 georeferenced points over the three vintages. Among 42 analytes, 22 were identified based on external standards (gallic acid (1), caffeic acid (2), *E*-resveratrol (3), *E*-piceatannol (4), catechin (5), epicatechin (6), gallocatechin (7), *E*-piceid (8), astilbin (10), *E*-ε-viniferin (13), *E*-δ-viniferin (15), quercetin-3-*O*-glucoside (16), ampelopsin A (17), quercetin-3-*O*-glucuronide (23), procyanidin B1 to B4 (24–27), *E*-miyabenol C (32), hopeaphenol (36), isohopeaphenol (37), and *Z*/*E*-vitisin B (41)). The 20 remaining compounds, including epicatechin-3-*O*-gallate (9); *Z*-resveratrol dimer 1 and 2 (12, 13); *E*-ω-viniferin (14); scirpusin A1 and A2 (18, 19); restrytisol A, B, and 3 (20–22); resveratrol dimer glycoside (28); α-viniferin (29); resveratrol trimer 1–3 (30, 31, 33); procyanidin trimer (34); dehydrogenated resveratrol tetramer (35); resveratrol tetramer 1–3 (38–40); and viniferol E (42), were tentatively assigned based on MS and UV spectra and elution order by comparison with literature (Table S1). PCA was performed to show similarities and differences among the polyphenol dataset of the three vintages. The PCA score plot of the two first components explained 62.3% of the variation (Figure 2A).

Centering and clustering of QC samples confirmed the robustness of UPLC-DAD-MS-based measurements and low analytical variability. Variation coefficients for each compound were less than 15% and 20% for metabolites with high and low concentrations, respectively, corresponding to standard precision of analytical methods [19]. The discrimination of the 3 years on PC1 showed a major vintage effect on total polyphenols (value = 17,352; *p* < 0.001; Table S2). Samples from 2015 and 2016 were projected on PC1 negative (low relative concentrations), while samples from the 2017 vintage were projected on PC1 positive with high relative concentrations for flavonoids and stilbenoid DP2, as shown by the corresponding loading plot (Figure 2B). PCA observations were completed by robust two-way ANOVA for trimmed means, with interaction effects showing significant vintage (*p* < 0.05) and spatial effects (*p* < 0.001), as well as their interactions (*p* < 0.001), for all compounds (Table S2).

Interestingly, intra-vintage variations were projected on the PC2 axis, showing that a subset of polyphenols including stilbenoid DP4 (dehydrogenated resveratrol tetramer (35), hopeaphenol (36), resveratrol tetramer 1 (38), resveratrol tetramer 3 (40), *Z*/*E*-vitisin B (41), and viniferol E (42)) as well as stilbenoid DP2 (ampelopsin A (17), scirpusin A1 (18), and restrytisol B (21)) were the main drivers of intra-vintage variation, suggesting a conserved effect, regardless of the year.

### 2.3. Intra-Vintage Variations in Metabolomics Data

**Figure 3:**
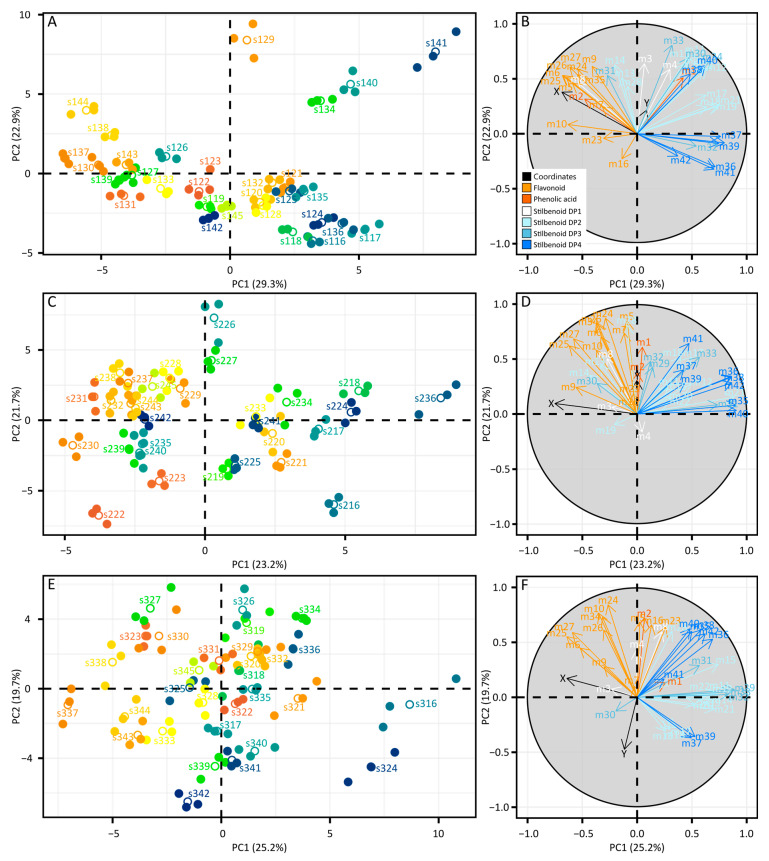
PCA of metabolomics data and geographic coordinates during 2015 (**A**,**B**), 2016 (**C**,**D**), and 2017 (**E**,**F**). Variables in score plots (**A**,**C**,**E**) are colored according to elevation. Variables in loading plots (**B**,**D**,**F**) are colored according to polyphenol classes and numbered according to the compound name given in Figure 2. Longitude and latitude coordinates (X, Y) are presented in black on loading plots (**B**,**D**,**F**).

PCAs were performed on data subsets corresponding to each single vintage. PCA on the 2015 dataset explained 52.2% of variation (Figure 3A).

The corresponding loading plot clearly showed that metabolites were projected on PC1 and PC2 according to polyphenol subclasses, with flavonoids and stilbenoids as the two main groups (Figure 3B). To assess potential correlations between metabolomics and soil variables, longitude and latitude coordinates (X, Y) were implemented. Samples projected on PC1 negative showed higher contents in flavonoids along with high X (longitude) values, i.e., in the east direction toward the top of the parcel. Conversely, samples projected on PC1 positive were characterized by high content in stilbenoid DP4, along with low X values, i.e., in the west direction down in the parcel. In complement to PCA, univariate statistics confirmed a spatial effect for all compounds (*p* < 0.001, Table S2). Similar analyses were performed on data subsets corresponding to subsequent vintages and, interestingly, the same trends were observed (Figure 3C–F). Flavonoid-rich samples were projected in PC2 negative together with longitude, corresponding to the top of the parcel in the east direction. Stilbenoid-DP4-rich samples were projected on PC1 positive opposite to X values, corresponding to the bottom of the parcel in the west direction. Univariate statistics also confirmed the spatial effect on each metabolite for these vintages (*p* < 0.001, Table S2). Besides the prominent vintage effect shown previously, these results showed a clear spatial effect on polyphenols from grape canes in relation to geographic coordinates.

### 2.4. Correlation-Based Metabolite Networks to Assess the Structuration of Polyphenol Metabolism with Topography and Soil Parameters

**Figure 4:**
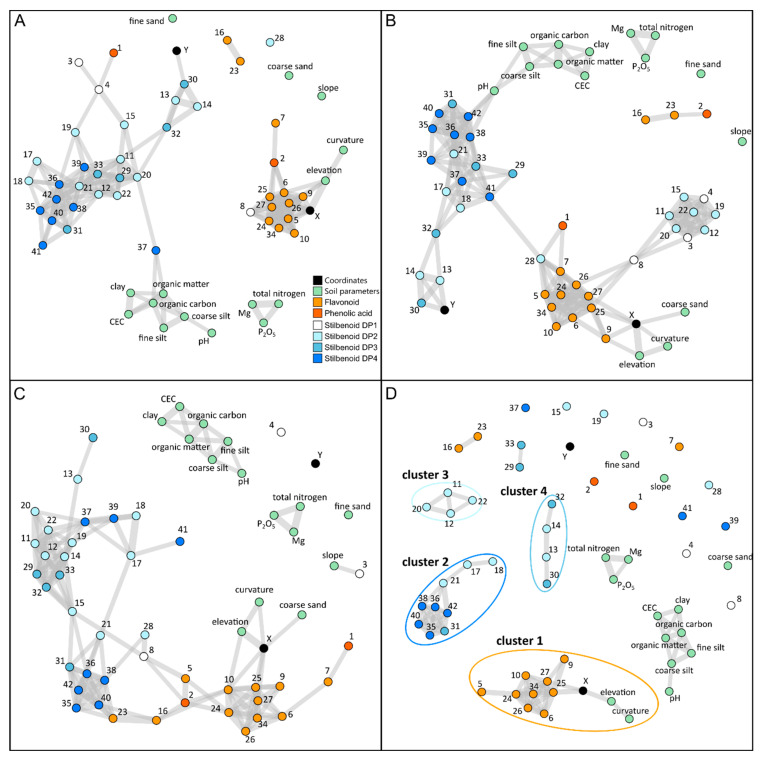
Correlation-based networks of metabolomics data in 2015 (**A**), 2016 (**B**), and 2017 (**C**) and conserved over 3 years (**D**) with soil parameters and geographical coordinates. Pairs whose correlation was significant with a minimum Pearson correlation coefficient of 0.5 are connected. Nodes are colored according to polyphenols’ class and numbered according to the compound name given in Figure 2. A short node distance indicates a high correlation.

First, attempts were made to spatialize single metabolites within the vineyard; however, the variability was too high to show any trend of spatialization. We used correlation networks to select co-varying metabolites for future spatialization studies. In this way, pair-wise Pearson correlations between 42 polyphenols, geographic coordinates, as well as topographic and soil parameters were calculated for each vintage dataset. Among the 1711 tested correlations, the metabolite network from vintage 2015 showed 165 significant positive correlations (threshold: r > 0.5; *p* < 0.05), the network from vintage 2016 showed 171 significant positive correlations (threshold: r > 0.5; *p* < 0.05), while the network from vintage 2017 encompassed 140 significant positive correlations (threshold: r > 0.5; *p* < 0.05). Figure 4A–C shows the corresponding networks with metabolite, topography, and soil parameters represented as nodes and significant correlations by edges. The shorter the node distance, the higher the correlation. Polyphenols were intercorrelated according to different subclasses, forming different metabolite clusters. Additionally, soil parameters formed two clusters, with the first composed of clay, fine silt, CEC, organic matter, organic carbon, coarse silt, and pH, and the second composed of total nitrogen, Mg, and P_2_O_5_. Whereas network structures varied over the 3 consecutive years in response to vintage effect, four metabolic clusters were observed regardless of the year. The first cluster was mainly composed of flavonoids, the second by stilbenoid DP2 and DP3, and the third by stilbenoid DP3 and DP4. To exclude correlations induced by vintage-specific environmental factors, a new network based only on conserved correlations was built, exhibiting 72 significant positive correlations (threshold: r > 0.5; *p* < 0.05) and four main metabolite clusters (Figure 4D). The first cluster was composed of flavonoids (cluster 1; catechin (5), epicatechin-3-*O*-gallate (9), astilbin (10), quercetin-3-*O*-glucoside (16), quercetin-3-*O*-glucuronide (23), procyanidin B1 (24), procyanidin B2 (25), procyanidin B3 (26), procyanidin B4 (27), and procyanidin trimer (34)) and the second of stilbenoid DP3 and DP4 (cluster 2; resveratrol trimer2 (31), dehydrogenated resveratrol tetramer (35), hopeaphenol (36), resveratrol tetramer1 (39), resveratrol tetramer3 (40), and viniferol E (42)). Additionally, two minor clusters were observed including a limited number of metabolites. Cluster 3 was composed of stilbenoid DP2 and DP3 (*E*-ε-viniferin (13), *E*-ω-viniferin (14), and resveratrol trimer 1 (30)) and cluster 4 was composed of stilbenoid DP3 (α-viniferin (29), *E*-miyabenol C (32), and resveratrol trimer 3 (33)). The repetitive occurrence of these clusters suggested control by environmental factors with lasting effects over vintages and probably in relation to topography and soil properties. The conserved metabolic cluster 1 presented edges with X (longitude), elevation, and curvature, suggesting that flavonoid metabolism was induced toward the east direction, where the slope and elevation are important. In contrast, no conserved correlations were found between the texture and the chemical composition of soils and the metabolic variables. From there, spatialization studies were conducted on these conserved metabolomics clusters.

### 2.5. Spatialization Studies of Metabolomics Clusters

**Figure 5:**
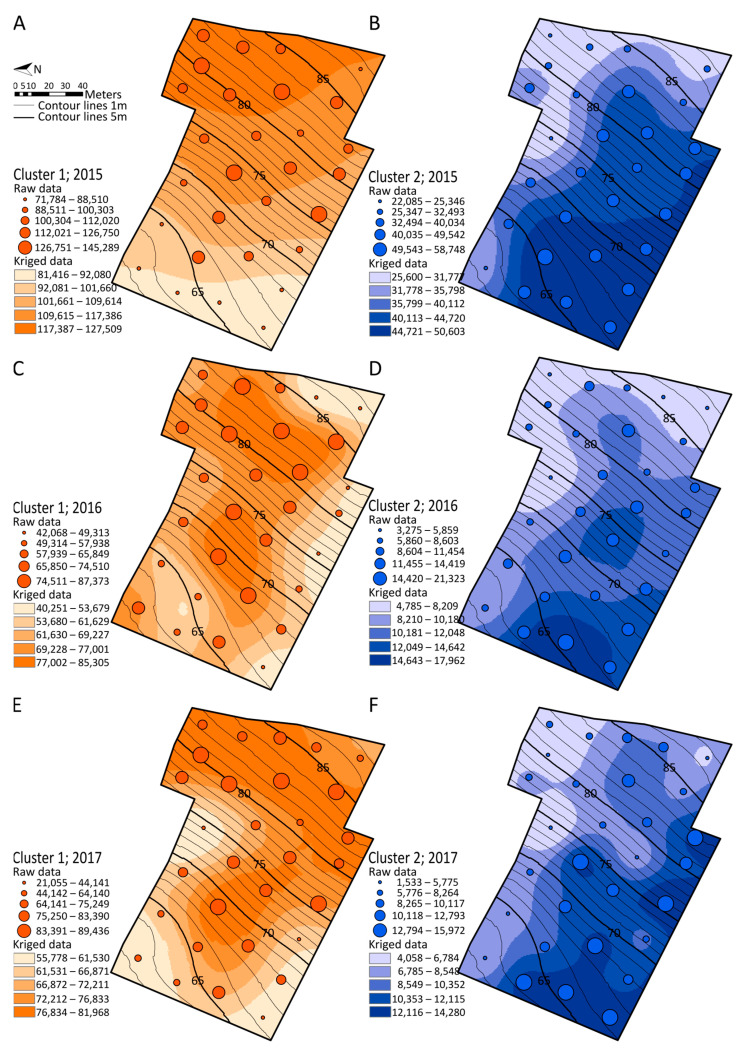
Spatial representation of conserved metabolic clusters in 2015 (**A**,**B**), 2016 (**C**,**D**), and 2017 (**E**,**F**). Cluster 1 composed of flavonoids (**A**,**C**,**E**). Cluster 2 composed of stilbenoid DP4 (**B**,**D**,**F**).

The sum of the relative abundances of metabolites comprised in the conserved metabolic clusters was spatialized using GPS positions and represented as circles of relative sizes on the maps (Figure 5). Maps of cluster 1, composed of flavonoids, showed a clear spatial effect over the three vintages, with the highest concentrations on top of the parcel and the lowest towards the bottom, with nearly a twofold amplitude (Figure 5A,C,E). Maps of cluster 2, composed of stilbenoid DP4, showed opposite trends of accumulation that were reproducible over the three vintages, with the lowest concentrations on the top of the parcel and the highest at the bottom of the parcel, representing a threefold amplitude (Figure 5B,D,F). A comparative analysis of maps from metabolic cluster 1 (Figure 5A,C,E) and from topography (Figure S2) showed that the relative concentrations of the selected metabolites were driven by changes in elevation and vineyard surface (slope and curvature). As a result, flavonoids were locally over-accumulated in sloping areas of the vineyard. In complement, the spatialization of metabolic clusters 3 and 4 was also performed (Figure S5). Although highly correlated, the spatialization of stilbenoid DP2 and DP3 (cluster 3) and stilbenoid DP3 (cluster 4) was highly variable depending on the vintage, and no correlation was found with either topography or soil properties.

## 3. Discussion

### 3.1. Experimental Design to Study Soil- and Topography-Based Terroir Influence

Advanced metabolomics methods combined with chemometrics have revealed terroir effects at regional and local levels in distant vineyards [14,15]. However, the terroir signature results from the complex interplay of multiple factors including grapevine genotypes (scion and rootstock), climate (temperature, rainfall, solar radiation, and biotic stresses), soil properties (pedology, geology, and microbiome), and topography, as well as viticultural and wine-making practices, and it remained difficult to unravel the potential significance of a single terroir component. The present study was designed in a continuous space of 1.35 ha with a spatial coverage sampling strategy and a focus on topography, soil properties, and grape cane metabolomic composition. Thanks to the E-Terroir Database, it was possible to select a vineyard with contrasted topography and soil properties, planted with a single clone Cabernet Franc grafted on the same rootstock and grown with the same viticultural practices. The present field setup was designed to mitigate at least some variations not studied herein, such as grapevine genetic and viticultural practices. However, in realistic vineyards conditions, it remains difficult to overcome significant vintage factors such as temperature, rainfall, solar radiation, and biotic stresses. A experimental design spanning several years could help to reveal the long-lasting vintage-specific effects.

### 3.2. Soil- and Topography-Based Terroir Signature Emerged behind the Vintage Effect

Multivariate statistical methods form valuable tools to integrate the complexity of multiparametric systems associated with the terroir concept. In the present study, PCA was used as an unbiased dimensionality-reduction method to summarize the variations within the 3-year dataset (Figure 2). The vintage effect (projected along PC1) was the first driver of grapevine metabolic variations, with higher polyphenol contents in 2017 compared with 2016 and 2015. Dry weather was observed in 2015 and 2016 (Figure S6) in contrast to regular rainfalls in 2017, and it is likely that climatic conditions in 2017 were suitable for fungal developments with potential induction on grape phytoalexins. It has been observed that drought conditions significantly reduced stilbene concentrations in wines [20] and, conversely, powdery and downy mildews increased stilbenoid levels in leaves and canes [9]. The spatial effect (projected along PC2) was the second driver of metabolomic variations (Figure 2). This was confirmed by the sample distribution in vintage-specific PCAs (Figure 3), where polyphenol subclasses were projected according to vineyard orientation in a repeatable manner over the 3 years. Additionally, robust two-way ANOVA showed that the spatial effect was lower than the vintage effect for most of the metabolites (Table S2). In different studies on vines or wines using multivariate statistics, the detection of terroir influence with long-lasting signatures emerged behind vintage variations. From berry metabolomes on volatile and non-volatile compounds, it was possible to discriminate different macro-zones in Italy behind a dominant vintage effect [12]. A metabolomics study from geographically close vineyards highlighted a strong vintage effect in wine, skin, and must, but revealed a terroir-related signature only in bottle-aged wine [13]. The predominance of the vintage effect on grape metabolism always makes it difficult to study the underlying environmental impacts. Advanced methods in data curation such as network analysis could be helpful to reveal metabolomic data structuration.

### 3.3. Correlation-Based Networks Give an Overview of Metabolomic Data Structuration in Response to Environmental Changes

Network analysis is a simple way to visualize metabolite correlations and allows complex dataset interpretation without a preliminary assumption of biosynthetic pathways, even in noisy datasets [18]. Metabolites are displayed as nodes connected by “links” showing molecular interactions in a biological system. Thereby, metabolic fluctuations and interdependencies may result from similar control mechanisms under endogenous or exogenous factors. In grapevine, network analysis has been increasingly used in different contexts, including berry development, genotype difference, and changes in environmental factors [21]. In the present study, network structures over the three vintages showed high connectedness between structure-related metabolites, especially within flavonoids and stilbenoids; however, variations could also be observed over the three vintages. Then, we used a conserved network to represent the structuration of polyphenol metabolism in response to topography and soil composition over the 3 years. Four main conserved metabolite clusters were observed, composed according to polyphenol subclasses. Interestingly, grape canes showed similar metabolite clusters under varying environmental factors (this study) and genetic conditions [2]. The opposite clustering of flavonoids and stilbenoids has been previously observed and may rely on biochemical basis. STS is the key enzyme of stilbenoids biosynthesis and CHS is the first enzyme of flavonoid metabolism; both enzymes belong to the same superfamily of type III polyketide synthases and compete for the same substrates, that is, *p*-coumaroyl-CoA and 3 units of malonyl CoA. In the present study, depending on topography, either STS- or CHS-derived polyphenols were accumulated.

These two competing pathways might result from a complex network of WRKY and MYB transcription factors including both repressors and activators in response to environmental cues, including biotic downy mildew infection, mechanical wounding, or exposure to UV-C radiations [22]. About 100 stilbenoids, with various degrees of polymerization, have been reported for the *Vitis* genus [23]; however, only little knowledge is available regarding biochemical oligomerization of resveratrol oligomers [24]. In grape leaves exposed to UV-pulse, resveratrol monomers (DP1) accumulated earlier than viniferins (DP2), showing a time-dependent accumulation of resveratrol polymers [25]. The high connectivity of structure-related polyphenols suggests common biosynthesis regulatory mechanisms. Moreover, the clustering according to stilbenoid polymerization degree suggests that specific oxidative polymerization enzymes controlled biosynthetic fluxes within stilbenoid metabolism. However, enzymatic oxidative polymerization of stilbenoids remains poorly understood, although several grapevine class III peroxidases forming δ-viniferin have recently been proposed [26]. Considering *E*-resveratrol clustering (metabolite 3 in Figure 4), it was striking to observe different positions in correlation-based metabolite networks over the 3 years. Indeed, *E*-resveratrol variations correlated with flavonoids and stilbenoids DP2 in 2015 and 2016 (Figure 4A,B), but not in 2017 (Figure 4C), depending on vintages with different climatic conditions. This likely indicates that the biosynthesis of the stilbene phytoalexin precursor *E*-resveratrol is controlled by numerous regulatory mechanisms, as assumed by the identification of 47 STS family genes under the regulation of multiple MYB and WRKY transcription factors that coordinate grape defense in response to multiple environmental factors [27,28].

### 3.4. Specific Metabolite Clusters Correlated with Topography but Not Soil Composition

Correlation network analysis together with comparative analysis of maps showed that part of polyphenol metabolism was correlated with the topography, but not strictly with soil properties. Flavonoids (cluster 1) were locally induced in sloping areas and at the top of the vineyard. In contrast, stilbenoid DP4 (cluster 2) showed an opposite pattern of spatialization (Figure 5), with an induction at the bottom of the vineyard. Interestingly, the gradients of both metabolic clusters were oriented west-south-west, together with the slope direction. Although no strict correlation was found between metabolic clusters and soil properties, the contrasted soil composition of the parcel (Figures S3 and S4) suggests that upstream sandy soils, poor in organic matter and nitrogen, could be associated with high amounts of flavonoids, whereas downstream, finer particle soils, corresponding to colluvial deposits rich in organic matter and nitrogen, could be associated with stilbenoid DP4 (Figures S3 and S4).

Although no studies investigated the influence of soil features and topography on bioactive polyphenols from grape canes, interesting knowledge emerged from terroir studies based on berry polyphenols. The highest anthocyanin concentrations were found in the vineyards at a higher elevation facing south-west [29]. Ripening of grape berry is favored in soils with a low available water capacity through global polyphenol induction in berry, particularly anthocyanins, proanthocyanins, and flavonoids [30,31,32,33,34]. Our results suggest that the topography influenced flavonoids (cluster 1) in an opposite manner to stilbenoid DP4 (cluster 2). Interestingly, the conserved metabolite clusters 3 and 4, composed of stilbenoid DP2 and 3, showed no variation according to topography or soil properties. Their spatial distribution was specific to vintages, suggesting a regulation of resveratrol dimer and trimers by climatic factors such as temperature, rainfall, solar radiation, and biotic stresses.

### 3.5. Mapping Metabolomics Data in Agronomic Studies

Different omics approaches were applied to assess vine and wine sensory attributes in relation to regional variations, and metabolomics became a prevalent technology used to study genome–environment interactions [15]. The complexity of the terroir signature has been successfully addressed using metabolomics in distant or close vineyards; however, multiple environmental factors occurred concurrently [12]. Thanks to the combination of metabolomics and spatialization studies, it was possible to tackle soil- and topography-based terroir influence on grape cane metabolism at the parcel scale. The correlation-network-driven approach was used to select relevant metabolite clusters prior to spatialization studies. A conserved network from three vintages was generated to remove vintage-specific correlations and to select clusters potentially related to variations in soil composition and topography. These prefiltering steps allowed the extraction of biologically relevant information and spatialization studies on denoised metabolomic datasets. The combination of spatial metabolomics with rapid soil-phenotyping tools such as electromagnetic induction (EMI) that permits to differentiate specific characteristic areas in vineyards could assist the exploration of the terroir concept in viticulture [35]. Aboveground, the combination of spatial metabolomics with non-invasive phenotyping methods such as unmanned aerial vehicle (UAV) remote sensing methods will be effective to develop precision agriculture [36]. Beyond viticulture, where terroir is a significant concept, metabolomics-correlation-based networks combined with geographical information systems may be used as an additional field-phenotyping tool for precision agriculture to improve crop quality [37].

## 4. Materials and Methods

### 4.1. Vineyard Features, Environmental Parameters, and Geo-Referencing

This study was conducted during vintages 2015, 2016, and 2017 on producing grapevines of a commercial vineyard in the Loire Valley Region (France; 47°8′15.54″ N, 0°14′46.12″ E) selected with the E-Terroir database (http://eterroir-techniloire.com/, accessed on 3 June 2023). The vineyard was planted with a single clone of *Vitis vinifera* L. cv Cabernet Franc grafted on same rootstock at a density of 10,000 vines. ha^−1^ with a spacing of 1 m (within row) × 1 m (between rows). Vines were grown with standard organic management practices. A temperate oceanic climate was observed in the Loire Valley region. In 2015, the average daily temperature was 17.9 °C from May to September (growing season), with a maximum of 36.4 °C and a minimum of 5.2 °C (Figure S6A). There was rainfall of 319 mm over 55 rainy days. In 2016, the average daily temperature during the growing season corresponded to 18.5 °C, with a maximum of 38.1 °C and a minimum of 2.6 °C. There was rainfall of 181 mm over 48 rainy days (Figure S6B). During 2017, from May to September, the average daily temperature was 18.8 °C, with a maximum of 37.2 °C and a minimum of 4.2 °C. Rainfalls during this period reached 253 mm over 60 rainy days (Figure S6C). Thirty sampling positions were determined throughout the vineyard using a spatial coverage strategy [38] (Figure 1). The sampling points were located in the field using a Trimble GeoExplorer XT^®^ GPS (Trimble Navigation Limited, Sunnyvale, CA, USA) with a sub metric accuracy.

### 4.2. Soil Material and Particle Size Analysis

Surface soil samples were collected in 2015 with a soil auger (0–30 cm depth), resulting in 500 g of soil uptake. All samples were dried at 40 °C and sieved through a 2 mm mesh. Particle size analysis was determined using sieves and the pipette methods [39], resulting in the determination of five size fractions (clay: 0–2, fine silt: 2–20, coarse silt: 20–50, fine sand: 50–200, and coarse sand: >200 µm). Soil chemical analyses were performed according to international standards methods: cation exchange capacity (CEC) cobaltihexammine (NF ISO 23470), organic matter (Ann; NF ISO 14235), organic carbon (NF ISO 13878), total nitrogen (Dumas, NF ISO 13878), pH (NF ISO 10390), magnesium cobaltihexammine (NF X31-130), and P_2_O_5_ (Olsen method).

### 4.3. Extraction, Analysis, and Identification of Metabolites

For each geo-referenced sampling point, five stalks on the six closest grapevines (30 stalks) within a distance of one meter were pruned in early December in 2015, 2016, and 2017. These grape canes were cut into 10 cm long sections and stored for 10 weeks at 20 °C in the dark to allow *E*-resveratrol and *E*-piceatannol biosynthesis [40]. Grape canes were firstly ground with a cooled analytical grinder (Ika-Werke, Staufen, Germany) and then with a cutting mill (Polymix PX-MFC 90 D, Kinematica AG, Malters, Switzerland) to obtain 1 mm sized particles. The powder was lyophilized and stored at −20 °C until the polyphenol extraction.

Stilbenoid extraction was performed according to Houillé et al. (2015) [40]. Briefly, 50 mg of dried powder was extracted in 1 mL of ethanol/water (60/40; *v*/*v*) by shaking for 30 min at 83 °C (Thermomixer Comfort, Eppendorf AG, Hamburg, Germany) and centrifuged at 18,000× *g* for 5 min. The extracts were stored at −20 °C prior to further analyses and extemporarily diluted (1:5) in the starting mobile phase (acetonitrile/water/formic acid, 5/95/0.1, *v*/*v*/*v*) prior to UPLC-DAD-MS analyses. Those analyses were performed using an ACQUITY™ Ultra Performance Liquid Chromatography coupled with a photo diode array detector (DAD) and a Xevo TQD mass spectrometer (Waters, Milford, MA, USA) equipped with an electrospray ionization (ESI) source controlled by MassLynx 4.1 software (Waters, Milford, MA). Analyte separation was performed on a Waters Acquity HSS T3 C18 column (150 mm × 2.1 mm, i.d. 1.8 µm) at a flow rate of 0.4 mL min^−1^ at 55 °C after injection of 5 µL of the sample. The mobile phase consisted of solvent A (0.1% formic acid in water) and solvent B (0.1% formic acid in acetonitrile), and a linear gradient from 5 to 60% of B in 18 min was employed to achieve the chromatographic separation. MS detection was performed in positive and negative modes. The capillary voltage was 3 kV and the cone voltages were 30 V and 60 V. The cone and desolvation gas flow rates were 60 and 800 L.h^−1^, respectively. Analyte identification was managed according to retention times, m/z values, and UV spectra by comparison to commercial standards, in-house purified compounds, and literature data.

### 4.4. Metabolomic Data Analysis

The UPLC-DAD-MS method targeting polyphenols from grape canes was achieved using the selected ion monitoring (SIM) mode and resulted in SIM chromatograms integrated through the QuanLynx 4.1 subroutine. Peak integration was performed using the ApexTrack algorithm with a mass window of 0.1 Da and a relative retention time window of 1 min, followed by Savitzky–Golay smoothing (iteration = 1, width = 1). Additionally, integrated peaks were visually controlled. The present UPLC-DAD-MS method allowed the relative quantification of 42 metabolites, including 2 phenolic acids, 12 flavonoids, and 28 stilbenoids with various degree of polymerization: 3 resveratrol monomers (DP1), 12 dimers (DP2), 5 trimers (DP3), and 8 tetramers (DP4) (Table S1). To ensure the robustness of UPLC-DAD-MS analyses and to prevent analytical variability, we prepared quality control (QC) samples representative of the three vintages and the 30 georeferenced positions. QC samples were injected 15 times before running samples to equilibrate the system, and then once every eight samples and again 15 times after the run to check for potential analytical drifts. Every pool of grape canes corresponding to a GPS position was extracted and analyzed three times, corresponding to 270 injected samples (3 vintages × 30 sampling positions × 3 extractions). All samples were randomly injected and QC samples were analyzed by unsupervised principal component analysis (PCA) to evaluate the reproducibility of the method [19,41].

### 4.5. Statistical Analysis

PCAs were performed using R (v3.2.2, R Core Team, 2017) and the “FactoMineR” package. Variables were mean-centered and unit-variance scaled prior to multivariate analysis. Soil textural parameters were analyzed by PCA with the “soiltexture” package using the USDA triangle. As normality (‘shapiro_test’ function) and variance homogeneity (‘levene_test’ function) were not achieved for some variables, robust two-way ANOVA for trimmed means (20%) was computed using the ‘t2way’ function of the “WRS2” package and enabled primary factor assessment (year and spatial effect) as well as their interaction (year–spatial interaction). Pair-wise Pearson correlations were calculated in R using the “cor” and the “cor.test” functions. Significant correlations (R > 0.5; *p* < 0.05) were visualized as network correlations using the “igraph” (v1.0.1) and “gplots” packages. The “intersection” function was used to generate a correlation-based network conserved over the three vintages. Conserved metabolomic modules were considered for spatialization analyses.

### 4.6. Spatialization

The georeferenced coordinates for soil and grape cane sampling were implemented into ArcGIS (v10.2.2; ESRI Inc., Redlands, CA, USA) including soil properties, topography, and the relative concentration of conserved metabolomic clusters.

### 4.7. Chemicals

Gallic acid (1), caffeic acid (2), *E*-resveratrol (3), *E*-piceatannol (4), catechin (5), epicatechin (6), gallocatechin (7), *E*-piceid (8), astilbin (10), quercetin-3-*O*-glucoside (16), and quercetin-3-*O*-glucuronide (23) were purchased from Sigma-Aldrich standards (St. Louis, MI, USA). Procyanidins B1 (24), B2 (25), and B3 (27) were purchased from Extrasynthèse (Genay, France). *E*-ε-viniferin (13), *E*-δ-viniferin (15), ampelopsin A (17), *E*-miyabenol C (32), hopeaphenol (36), isohopeaphenol (37), and *Z*/*E*-vitisin B (41) were obtained as previously described [2]. Acetonitrile, methanol, and formic acid were purchased from ThermoFisher Scientific (Courtaboeuf, France). Ultra-pure water was prepared with a Milli-Q water purification system (Merck Millipore, Molsheim, France).

## 5. Conclusions

Metabolomics-correlation-based networks combined with GIS mapping were used as an unprecedented strategy to spatialize field-omics data and to explore soil- and topography-based terroir influence. Using multivariate statistics, terroir influence with long-lasting signatures emerged behind vintage variations. Correlation networks were successful to select relevant metabolite clusters prior to spatial distribution analyses. Only part of the polyphenol metabolism mirrored variations in topography with flavonoid metabolism induced upslope and resveratrol tetramers over-accumulated downslope. Spatial metabolomics driven by correlation-based networks thus represents a powerful approach for terroir zoning in viticulture and, more broadly, may serve as a new field-phenotyping tool in precision agriculture.

## Data Availability

All of the data supporting the findings of this study are included in this article.

## Supplementary Materials

The following supporting information can be downloaded at: https://www.mdpi.com/article/10.3390/molecules28114555/s1. Figure S1: Soil particles’ proportion as global USDA texture triangle (A) and with a focus on the sandy soil part (B). Sampling points were projected as colored circles. Cl: clay, Sa: sand, Si: silt, Lo: loam. Figure S2. Spatial representations of topographic variations. Figure S3: Spatial representation of the soil texture and the cation exchange capacity. Figure S4: Spatial representation of the soil chemical properties. Figure S5: Spatial representation of conserved metabolic clusters in 2015 (A,B), 2016 (C,D), and 2017 (E,F). Cluster 3 composed of stilbenoid DP2 and DP3 (A,C,E). Cluster 4 composed of stilbenoid DP3 (B,D,F). Figure S6: Vineyard climatic conditions during the 2015 (A), 2016 (B), and 2017 (C) growing seasons from May to September including daily minimal, average and maximal temperature (°C), and daily rainfall (mm). Table S1: List of grape cane polyphenols identified in the Cabernet Franc clone. Table S2: Non-parametric univariate statistics on vintage and spatial location over the 3 years of study and on spatial location into vintage.

## Sample Availability

Samples are available from the authors.
