# Supplementary material for: Terroir Influence on Polyphenol Metabolism from Grape Canes: A Spatial Metabolomic Study at Parcel Scale
Source: Molecules. 2023 Jun 5;28(11):4555. doi: 10.3390/molecules28114555 (PMC10254142; doi:10.3390/molecules28114555)
Supplement: Supplementary file 1 [file molecules-28-04555-s001.zip › molecules-2420030-supplementary.pdf]

molecules

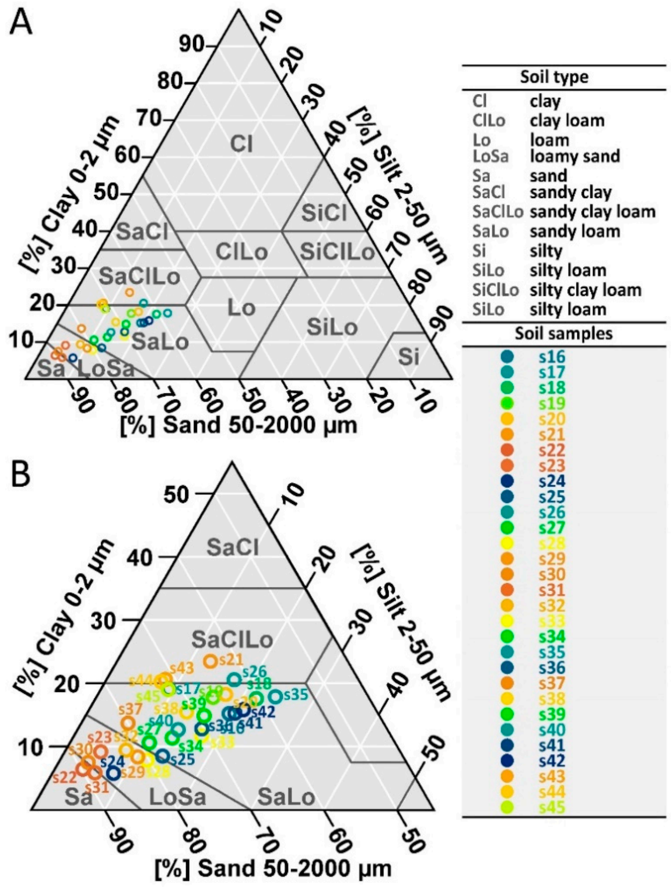

Figure S1. Soil particles proportion as global USDA texture triangle (A) and with a focus on sandy soil part (B). Sampling points were projected as colored circles. Cl: Clay, Sa: Sand, Si: Silt, Lo: Loam.

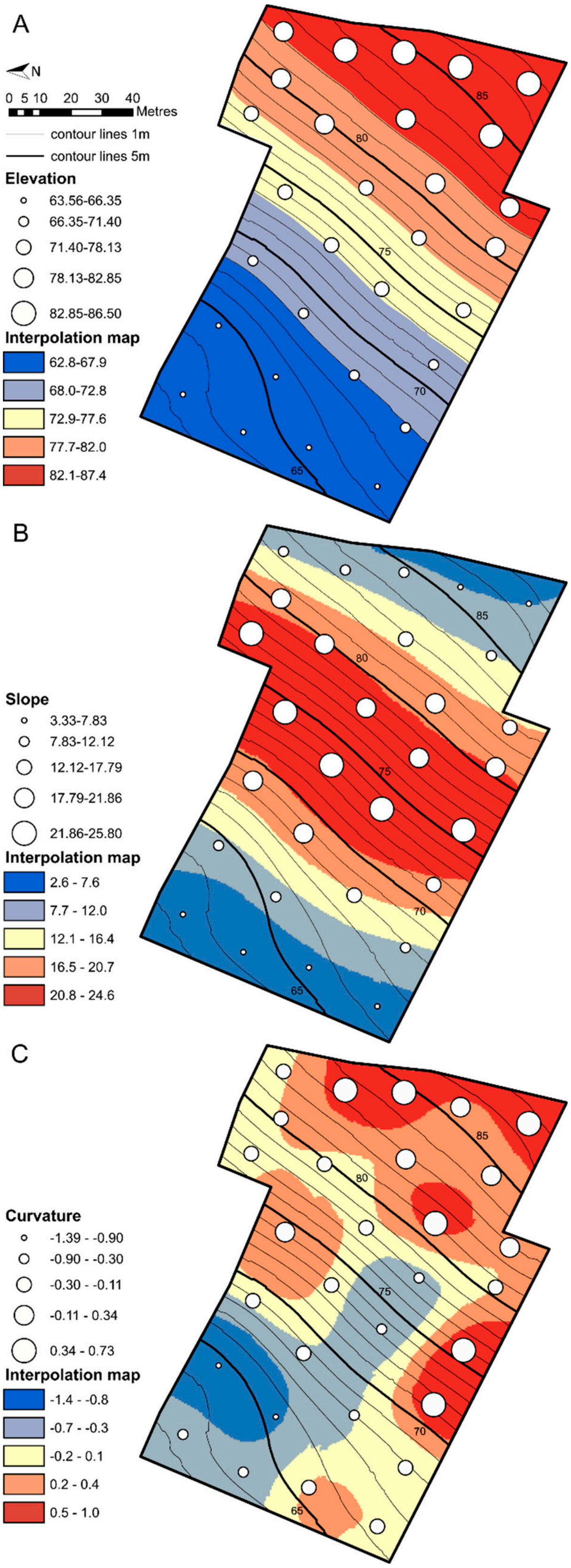

Figure S2. Spatial representations of topographic variations.

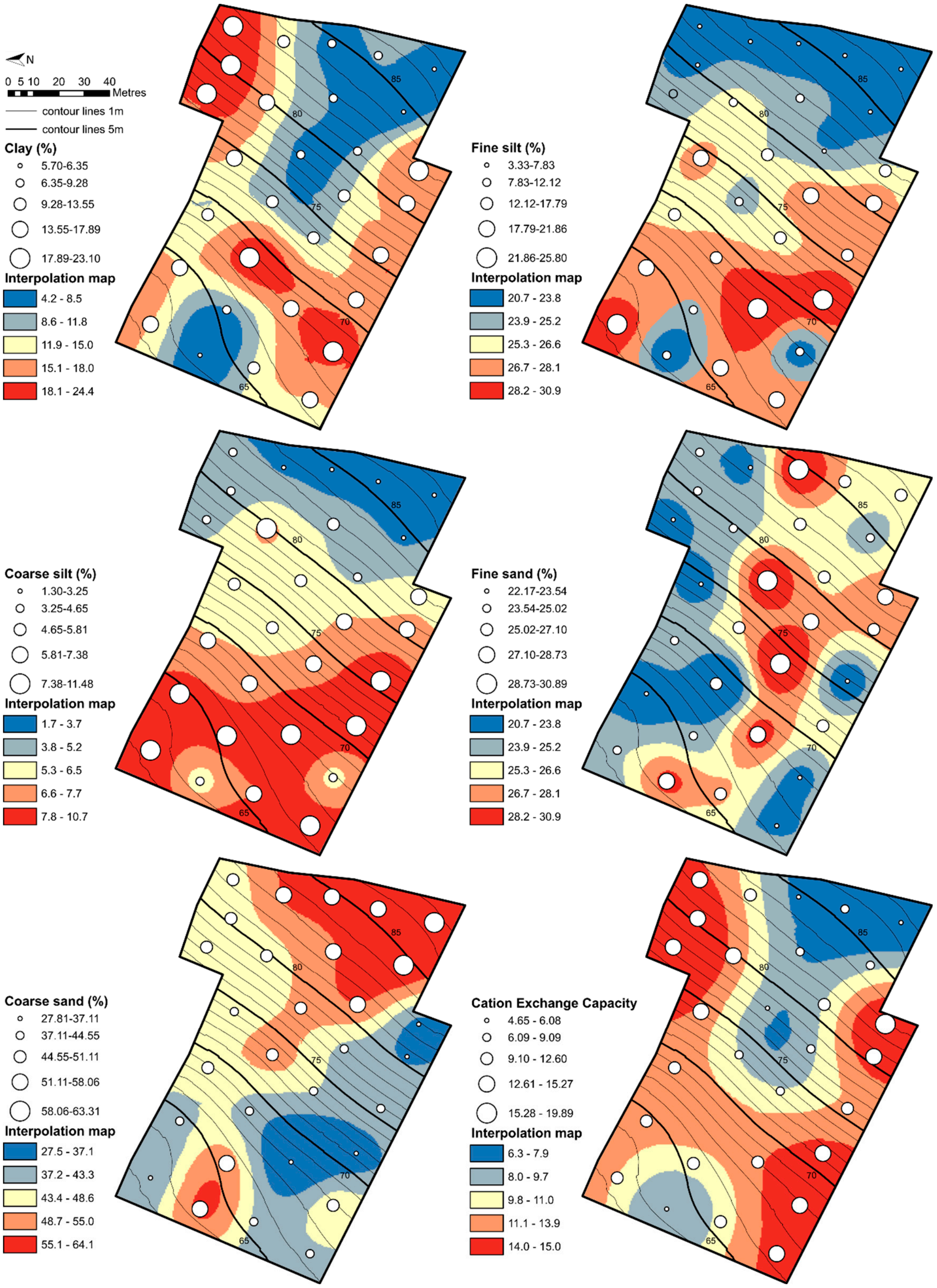

Figure S3. Spatial representation of the soil texture and the cation exchange capacity.

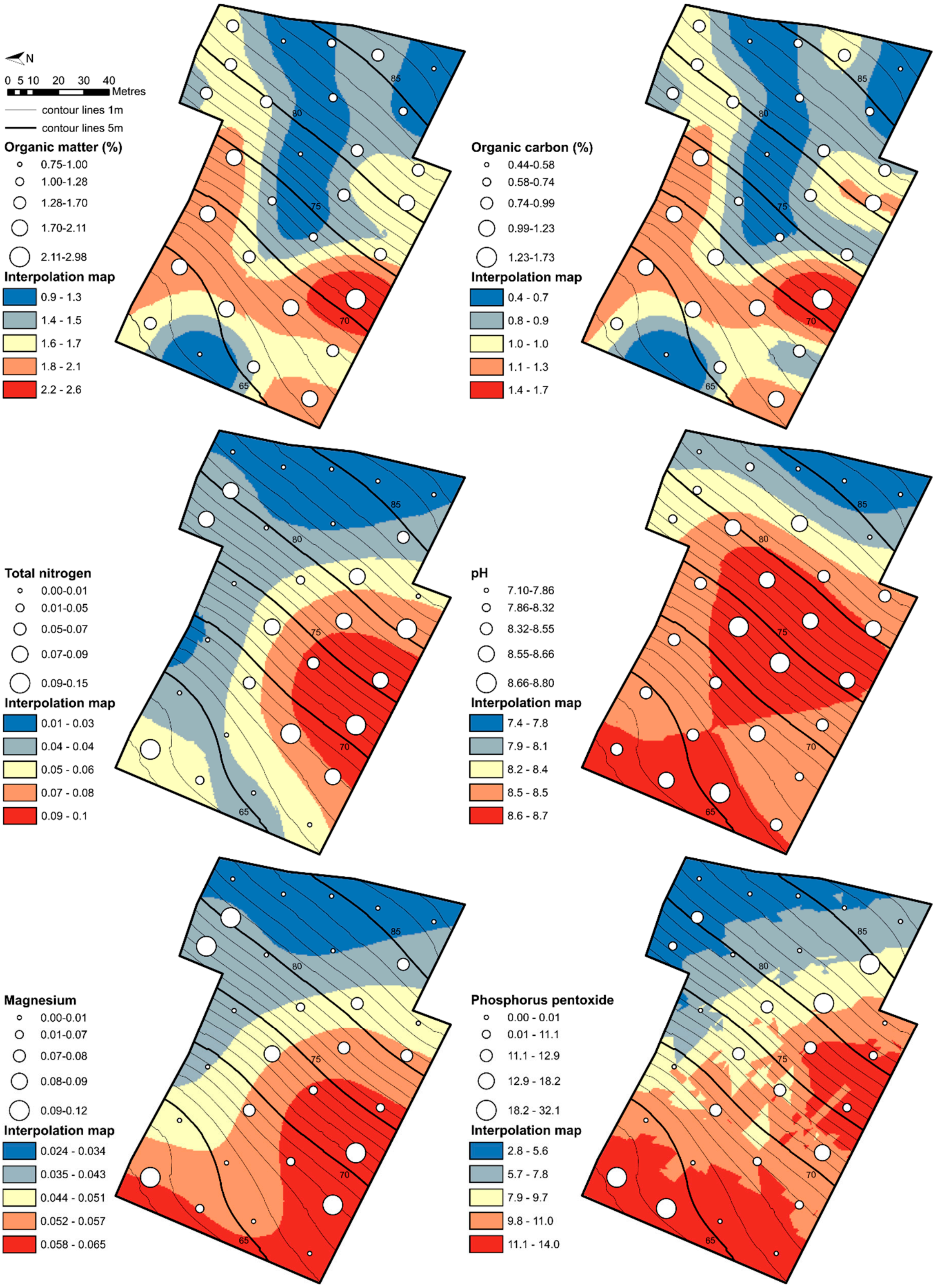

Figure S4. Spatial representation of the soil chemical properties.

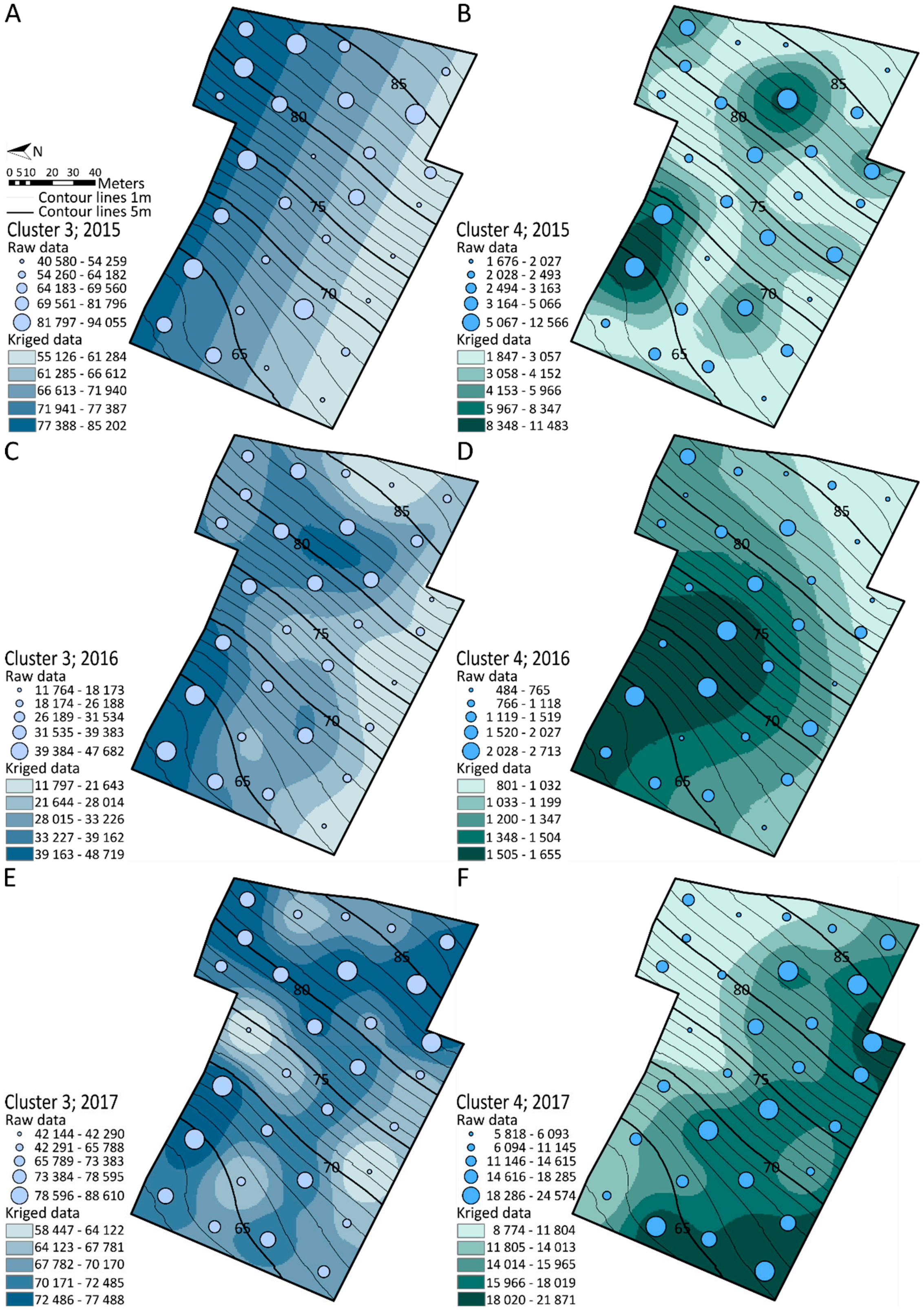

Figure S5. Spatial representation of conserved metabolic clusters in 2015 (A, B), 2016 (C, D) and 2017 (E, F). Cluster 3 composed of stilbenoid DP2 and DP3 (A, C, E). Cluster 4 composed of stilbenoid DP3 (B, D, F).

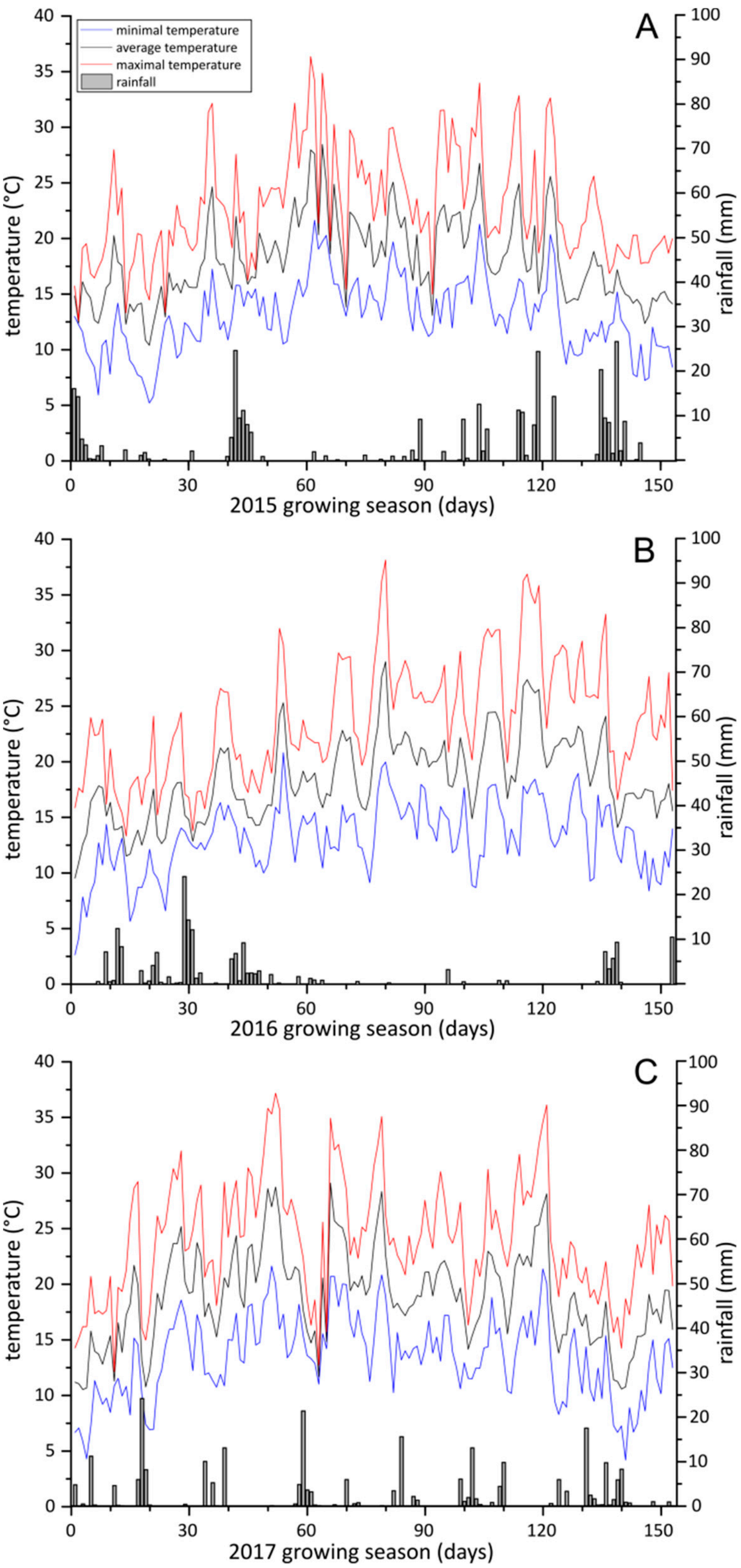

Figure S6. Vineyard climatic conditions during the 2015 (A), 2016 (B) and 2017 (C) growing seasons from May to September as daily minimal, average and maximal temperature (°C) and daily rainfall (mm).

Table S1. List of grape cane polyphenols identified in the Cabernet-Franc clone.

Peak	RT (min)	Phenolic class	Compound assignment	m/z [M-H]-	Product ions (ES-)	m/z [M+H]+	λmax (nm)	Authentication
1	1.91	Phenolic acid	gallic acid	169	79, 97, 78		269	Standard
2	3.22	Phenolic acid	caffeic acid	179	135, 79		327	Standard
3	9.56	Stilbenoid DP1	*E*-resveratrol	227	143, 185	229	305, 317	Standard
4	7.84	Stilbenoid DP1	*E*-piceatannol	243	159, 201	245	322	Standard
5	4.26	Flavonoid	catechin	289	203, 123	291	229, 278	Standard
6	5.39	Flavonoid	epicatechin	289	203, 123, 109	291	229, 278	Standard
7	3.81	Flavonoid	gallocatechin	305	125, 179	307	332, 369	Standard
8	8.77	Stilbenoid DP1	*E*-piceid	389	227, 185		278,7	Standard
9	7.27	Flavonoid	epicatechin-3-*O*-gallate	441	289, 169, 125	443	276,7	Vrhovsek *et al*., 2012; Ehrhardt *et al*., 2014
10	7.88	Flavonoid	astilbin	449		451	231, 289.7	Standard
11	8.96	Stilbenoid DP2	*Z*-resveratrol dimer1	453	277, 265	455	232sh, 279, 285	Püssa *et al*., 2006
12	11.71	Stilbenoid DP2	*Z*-resveratroldimer2	453	428	455	225, 282.7	Püssa *et al*., 2006
13	12.07	Stilbenoid DP2	*E*-ε-viniferin	453	347, 359, 225	455	225sh, 323	Standard
14	12.97	Stilbenoid DP2	*E*-ω-viniferin	453	347, 359, 225	455	225sh, 323	Moss *et al*., 2013
15	13.52	Stilbenoid DP2	*E*-δ-viniferin	453	347, 359, 225	455	225sh, 309	Standard
16	7.73	Flavonoid	quercetin-3-*O*-glucoside			465		Standard
17	7.84	Stilbenoid DP2	ampelopsin A	469	451, 375, 363	471	281,7	Standard
18	9.50	Stilbenoid DP2	scirpusin A1	469	451, 395	471	324,7	Moss *et al*., 2013, Shao *et al*., 2007
19	10.86	Stilbenoid DP2	scirpusin A2	469	379, 301	471	321,7	Moss *et al*., 2013, Shao *et al*., 2007
20	6.33	Stilbenoid DP2	restrytisol A	471	377, 246		230, 276	Cichewicz *et al*., 2000
21	7.15	Stilbenoid DP2	restrytisol B	471	379, 349		235, 267	Cichewicz *et al*., 2000
22	8.52	Stilbenoid DP2	restrytisol 3	471	389, 227		234, 330	Cichewicz *et al*., 2000
23	7.60	Flavonoid	quercetin-3-*O*-glucuronide	477	301, 151	479	256, 354	Standard
24	3.81	Flavonoid	procyanidin B1	577	289, 425, 407		280, 313	Standard
25	6.20	Flavonoid	procyanidin B2	577	289, 425, 407			Standard
26	4.12	Flavonoid	procyanidin B3	577			280, 313	Standard
27	4.86	Flavonoid	procyanidin B4	577	289, 425, 407			Standard
28	10.36	Stilbenoid DP2	resveratrol dimer glycoside	615	453, 359		323	Püssa *et al*., 2006; Moss *et al*., 2013
29	12.99	Stilbenoid DP3	α-viniferin	677	423	679	230sh, 285	Mattivi *et al*., 2011
30	10.36	Stilbenoid DP3	resveratrol trimer 1	679	585, 491	681		Püssa *et al*., 2006
31	10.76	Stilbenoid DP3	resveratrol trimer 2	679	585, 491	681		Püssa *et al*., 2006
32	12.39	Stilbenoid DP3	*E*-miyabenol C	679	573, 451	681	284	Standard
33	12.74	Stilbenoid DP3	resveratrol trimer 3	679	585, 491			Püssa *et al*., 2006
34	4.38	Flavonoid	procyanidin trimer	865	664, 576	867	279	Monagas *et al*., 2006
35	12.69	Stilbenoid DP4	dehydrogenated resveratrol tetramer	904	679		295	Ito *et al*., 1999

36	10.65	Stilbenoid DP4	hopeaphenol	905	811, 717, 705, 451, 359	907	281,6	Standard
37	10.94	Stilbenoid DP4	isohopeaphenol	905	811, 717, 451, 359	907	283,9	Standard
38	11.47	Stilbenoid DP4	resveratrol tetramer 1	905		907	285	Püssa *et al.*, 2006, Flamini *et al.*, 2013
39	11.86	Stilbenoid DP4	resveratrol tetramer 2	905		907	284, 331	Püssa *et al.*, 2006, Flamini *et al.*, 2013
40	12.68	Stilbenoid DP4	resveratrol tetramer 3	905	573	907	323	Püssa *et al.*, 2006, Flamini *et al.*, 2013
41	14.32	Stilbenoid DP4	*Z*/*E*-vitisinB	905	799, 359	907	322	Standard
42	10.40	Stilbenoid DP4	viniferol E	923				Fujii *et al.*, 2005, Rivière *et al.*, 2010

Table S2. Non-parametric univariate statistics on vintage and spatial location over the 3 years study and on spatial location into vintage.

		Year effect			Spatial effect			Year-Spatial interaction		
Peak	Compound assignment	value	p-value		value	p-value		value	p-value	
1	gallic acid	523	0.001	***	326	0.001	***	768	0.001	***
2	caffeic acid	10989	0.001	***	3322	0.001	***	7801	0.001	***
3	*E*-resveratrol	60519	0.001	***	3949	0.001	***	83646	0.001	***
4	*E*-piceatannol	49504	0.001	***	2723	0.001	***	144193	0.001	***
5	catechin	2739	0.001	***	4213	0.001	***	5693	0.001	***
6	epicatechin	5265	0.001	***	6796	0.001	***	4677	0.001	***
7	gallocatechin	8156	0.001	***	7339	0.001	***	13479	0.001	***
8	*E*-piceid	28130	0.001	***	13128	0.001	***	46391	0.001	***
9	epicatechin-3-*O*-gallate	9154	0.001	***	9532	0.001	***	6992	0.001	***
10	astilbin	413	0.001	***	4310	0.001	***	4832	0.001	***
11	*Z*-resveratrol dimer1	18403	0.001	***	16043	0.001	***	54679	0.001	***
12	*Z*-resveratroldimer2	11819	0.001	***	16561	0.001	***	30663	0.001	***
13	*E*-ε-viniferin	3483	0.001	***	5691	0.001	***	4552	0.001	***
14	*E*-ω-viniferin	12763	0.001	***	29155	0.001	***	49289	0.001	***
15	*E*-δ-viniferin	15169	0.001	***	8070	0.001	***	23760	0.001	***
16	quercetin-3-*O*-glucoside	141	0.001	***	1985	0.001	***	4993	0.001	***
17	ampelopsin A	1600	0.001	***	1934	0.001	***	4584	0.001	***
18	scirpusin A1	1068	0.001	***	963	0.001	***	3044	0.001	***
19	scirpusin A2	9819	0.001	***	4645	0.001	***	13001	0.001	***
20	restrytisol A	29229	0.001	***	22672	0.001	***	59611	0.001	***
21	restrytisol B	931	0.001	***	1757	0.001	***	4557	0.001	***
22	restrytisol 3	11082	0.001	***	3899	0.001	***	45796	0.001	***
23	quercetin-3-*O*-glucuronide	208	0.001	***	1057	0.001	***	8253	0.001	***
24	procyanidin B1	1600	0.001	***	2958	0.001	***	4653	0.001	***
25	procyanidin B2	6397	0.001	***	4829	0.001	***	6977	0.001	***
26	procyanidin B3	3317	0.001	***	2107	0.001	***	2413	0.001	***
27	procyanidin B4	9537	0.001	***	5349	0.001	***	4050	0.001	***
28	resveratrol dimer glycoside	3021	0.001	***	4516	0.001	***	5416	0.001	***
29	α-viniferin	11975	0.001	***	93817	0.001	***	378207	0.001	***
30	resveratrol trimer 1	165	0.001	***	22515	0.001	***	20492	0.001	***
31	resveratrol trimer 2	497	0.001	***	3178	0.001	***	3967	0.001	***
32	*E*-miyabenol C	10074	0.001	***	39680	0.001	***	55915	0.001	***
33	resveratrol trimer 3	11784	0.001	***	13752	0.001	***	29938	0.001	***
34	procyanidin trimer	1378	0.001	***	1323	0.001	***	2113	0.001	***
35	dehydrogenated resveratrol tetramer	357	0.001	***	7367	0.001	***	16547	0.001	***
36	hopeaphenol	13	0.003	**	1527	0.001	***	4791	0.001	***
37	isohopeaphenol	7543	0.001	***	8290	0.001	***	22668	0.001	***
38	resveratrol tetramer 1	7	0.042	*	1506	0.001	***	3180	0.001	***
39	resveratrol tetramer 2	8979	0.001	***	7698	0.001	***	13346	0.001	***
40	resveratrol tetramer 3	392	0.001	***	7390	0.001	***	11943	0.001	***
41	*Z*/*E*-vitisinB	663	0.001	***	1846	0.001	***	14696	0.001	***
42	viniferol E	46	0.001	***	2602	0.001	***	6804	0.001	***
	Total polyphenols	17352	0.001	***	3597	0.001	***	5611	0.001	***
